# A Case of Metastatic Melanoma Post Orthotopic Liver Transplantation

**DOI:** 10.7759/cureus.60347

**Published:** 2024-05-15

**Authors:** Samantha Ortiz Muriel, Rahed Mohammed, Kathryn Bass, Prathima Gopinath, Anthony Manasia

**Affiliations:** 1 Institute for Critical Care Medicine, Mount Sinai Hospital, New York, USA

**Keywords:** peri-transplant screening guidelines, immunosuppressive therapy, post-transplantation melanoma, olt, orthotopic liver transplantation

## Abstract

With the rising prevalence of organ transplantation, clinicians must be aware of the many potential complications that may arise. One such complication is post-transplantation melanoma. Herein, we present a case of advanced metastatic melanoma following orthotopic liver transplantation (OLT).

This is a 54-year-old cirrhotic male who underwent OLT that was complicated by metastatic melanoma. Despite adherence to yearly screening guidelines and timely radiation and immunotherapy, the disease course was rapidly progressive and fatal. This case aims to highlight the risk of post-transplantation melanoma and the potential need for screening modifications to identify melanoma earlier in its development.

The association between organ transplantation and melanoma is well-reported, but the underlying risks and mechanisms remain incompletely understood. One potential risk factor is post-transplant immunosuppressive therapy, which may result in fatally aggressive melanoma. Understanding the potential mortality risks in transplant patients, modifications to peri-transplant screening guidelines, and immunosuppressive therapy may be lifesaving.

## Introduction

According to the United Network for Organ Sharing (UNOS) database, 42,887 organ transplants were performed in the United States in 2022 [1]. This was a 3.7% increase from the previous year [1]. With a steady rise in the number of patients receiving organ transplantation, it is important that clinicians recognize and understand the many potential complications that may arise [2,3]. To date, several studies report an increasing incidence of melanomatous skin cancer following organ transplantation, which is reportedly 1.8-8 times greater than that of the general population [4,5]. Studies have also reported a threefold increased mortality rate in post-transplant patients compared to non-transplant recipients [6]. While factors such as increasing age, race and ethnicity, and immunosuppression have been shown to increase the risk of post-transplant melanoma, there are likely many other factors affecting its incidence [7]. Although screening guidelines exist to help identify patients at high risk for melanoma, it is unclear whether these guidelines are sufficient in very-high-risk patients with multiple risk factors. Herein, we present a case of post-liver transplantation melanoma that recurred and aggressively metastasized despite adherence to guideline-based screening intervals.

## Case presentation

This is a 54-year-old male with a history of alcoholic cirrhosis who underwent orthotopic liver transplantation (OLT) in 2017. The patient's post-operative course was complicated by melanoma that was surgically removed but aggressively recurred despite guideline-driven melanoma screening.

In 2017, the patient underwent an uncomplicated OLT and was started on tacrolimus, mycophenolate, and prednisone for immunosuppression. Due to post-operative encephalopathy, tacrolimus was switched to cyclosporine. Following this change, the patient’s encephalopathy improved, and he was discharged home two weeks following the transplant.

In August 2020, the patient's primary care physician identified a lesion on the right chest wall. The lesion appeared asymmetric, had irregular borders, and measured 1.5 cm in diameter. Biopsy was performed and histologically revealed an ulcerated proliferation of atypical melanocyte units and nests within the epidermis and extending deep into the reticular dermis. Numerous atypical mitotic figures were found within the dermal component. Based on this pathology, a diagnosis of stage IIA malignant melanoma was established. The patient was referred to surgical oncology, and a wide-margin surgical excision with right axillary sentinel lymph node biopsy was performed. The excised lesion extended 1.9 mm below the skin surface, consistent with a Breslow T2 classification. There was no evidence of lymphovascular invasion, and the lymph node biopsy was negative for tumor. No further therapy was indicated, and based on guidelines, the patient was closely followed with yearly outpatient skin examinations. There was no indication for surveillance imaging, and there were no modifications made to the patient's immunosuppressive regimen.

**Figure 1:**
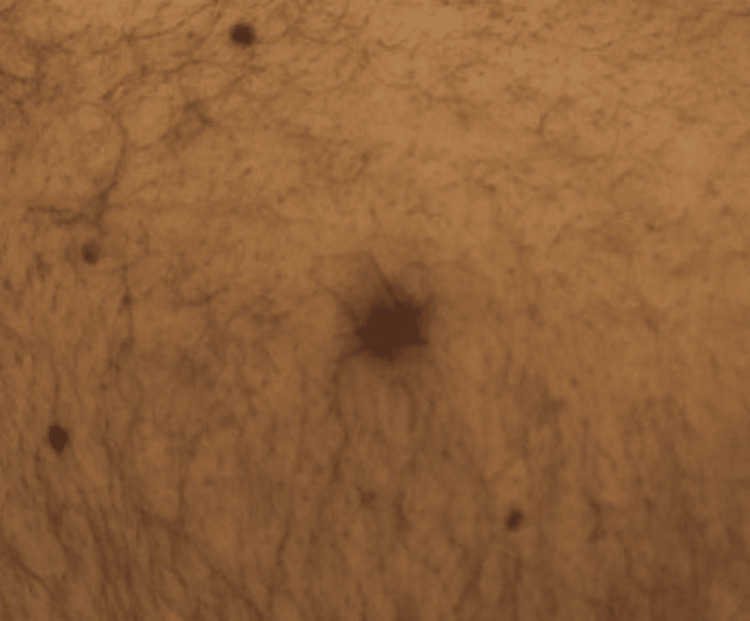
Pigmented skin lesions characterized by irregular borders.

In 2021, the patient followed up with his annual surveillance skin examination, which did not reveal any concerning lesions. During the patient's annual examination in September 2022, several extensive skin lesions were detected by outpatient dermatology. These lesions were pigmented and characterized by irregular borders (Figure 1).

**Figure 2:**
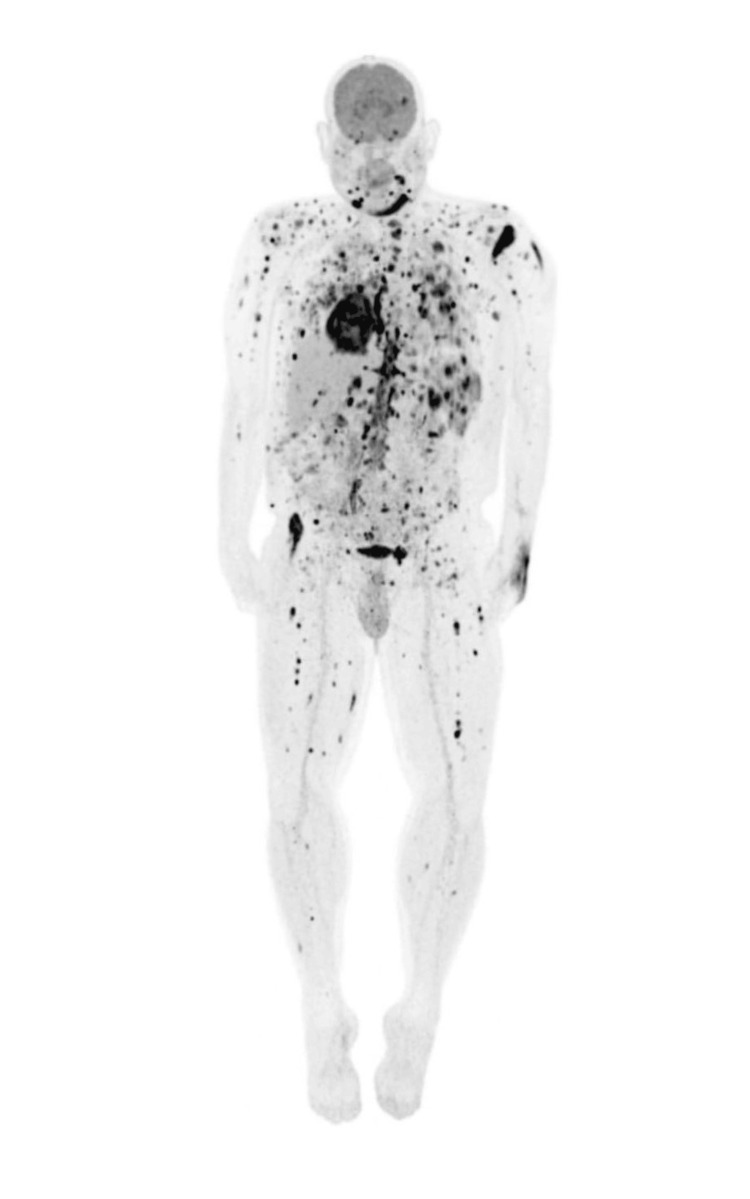
Scanning PET/CT scan. PET/CT showing innumerable hypermetabolic lesions in the cutaneous surfaces with multiple hypermetabolic osseous and intramuscular lesions throughout the axial and appendicular regions. Additionally, there are hypermetabolic lesions in the brain, thoracic and lumbar spine, pulmonary tissue, salivary glands, heart, bowels, liver, spleen, bilateral adrenal glands, pancreas, and peritoneum and throughout the abdominal wall.

Biopsies were sampled from multiple sites. Shave biopsy taken from the scalp histologically demonstrated nests of atypical cells with variable nuclear size and occasional mitotic figures. Punch biopsy from the patient's left shoulder histologically revealed a dense nodule of markedly atypical cells with variable nuclear size and occasional mitotic figures. Both of these biopsy samples stained strongly positive for SOX-10 but negative for ALK-1 and CAM5.2. Staging PET/CT revealed extensive intra-cranial and extra-cranial lesions involving innumerable anatomic regions, which was consistent with diffuse metastatic disease (Figure 2).

**Figure 3:**
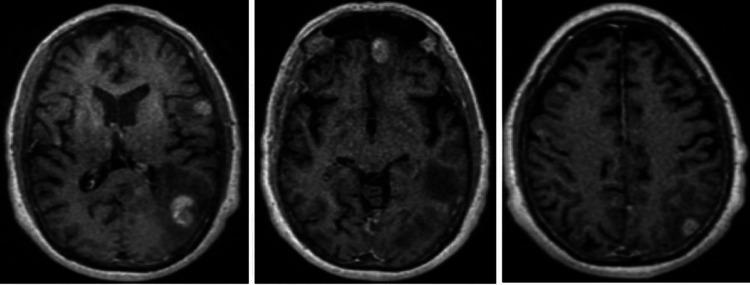
Brain MRI revealing multiple heterogeneously enhancing metastatic lesions.

Additionally, brain MRI revealed multiple heterogeneously enhancing hemorrhagic lesions, consistent with metastatic disease (Figure 3).

Multiple additional skin sites and a lumbar spine lesion were also biopsied. Histology was similar to that described above, and in addition to SOX-10, staining was also strongly positive for melan-A and S-100 (though negative for BRAF and VE-1). Given this constellation of features, the patient was diagnosed with stage IV metastatic melanoma. Systemic corticosteroids combined with brain and spine radiation were immediately started. Additionally, nivolumab and relatlimab were added to the immunosuppressive regimen.

**Figure 4:**
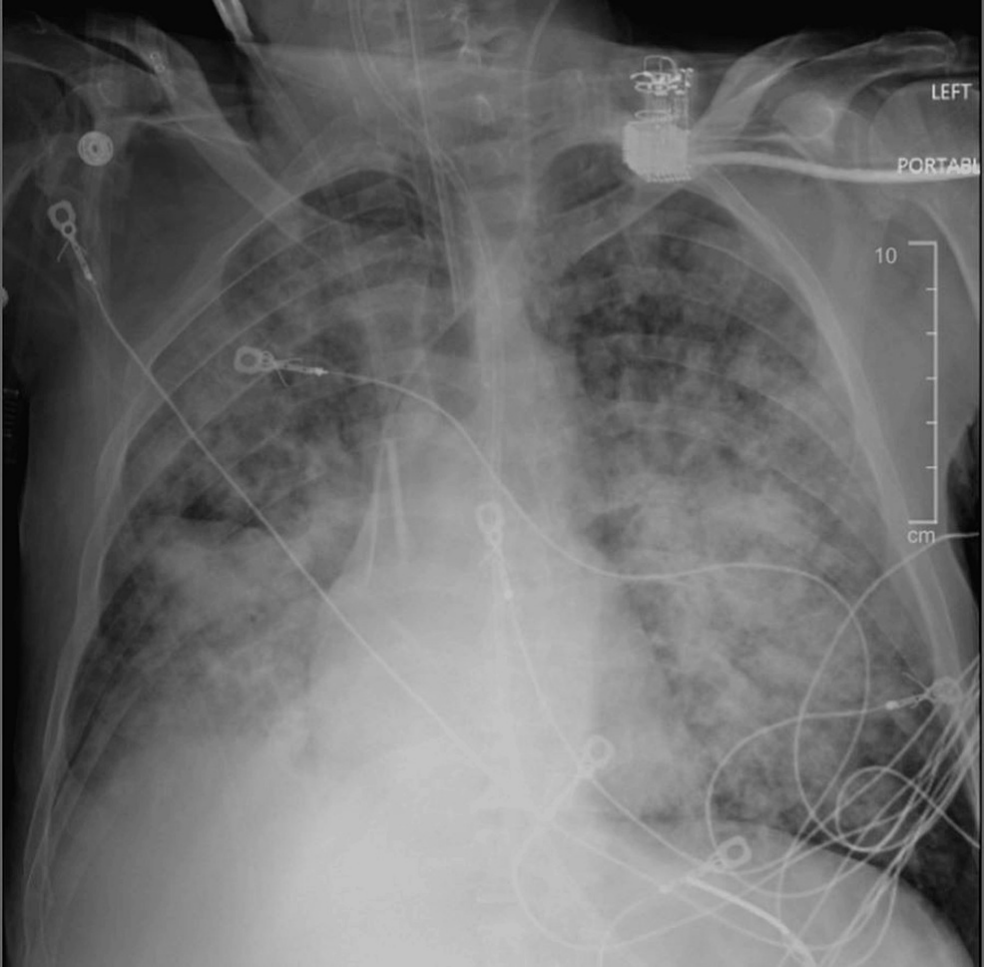
Chest X-ray revealing diffuse pulmonary opacities.

Despite aggressive therapy, the patient developed generalized and unremitting pain, anorexia, nausea, vomiting, and agitation over the subsequent three months. In January 2023, the patient was admitted to the hospital after presenting with frank hemoptysis. Within one day of admission, acute hypoxemic respiratory failure ensued, and imaging revealed diffuse pulmonary opacities (Figure 4).

Encephalopathy developed shortly afterwards, resulting in intubation and transfer to the intensive care unit (ICU). The ICU course was complicated by multi-organ failure, which included acute renal and liver failure. Given these circumstances, the decision was made to transition the patient to a comfort-focused model of care. All lifesaving measures were deferred, and the patient was transferred to the palliative care unit. Within one day of transfer, the patient expired. Informed consent was obtained from the patient's healthcare proxy for case report publication.

## Discussion

It is well-documented that the prevalence of melanoma following organ transplantation exceeds that of the general population [8]. One of the largest studies examining organ transplantation found a 2.38-fold increase in the prevalence of melanoma when compared to the general population [5,9]. However, there remains a poor overall understanding of the mechanisms underlying the development of malignant melanoma in transplant recipients. Additionally, some studies report that transplant recipients who develop melanoma are more likely to present with higher Breslow thickness scores, suggesting an increase in both the prevalence and aggressiveness of post-transplant melanoma [10]. It is not entirely clear why the aggressiveness of post-transplant melanoma exceeds what is typically observed in the non-transplant population. According to the literature, mortality can range from 5% to 50%, and transplant recipients who present with higher Breslow thickness scores experience worse clinical outcomes [11]. Unfortunately, there are no validated models that can reliably predict aggressiveness or prognosis in those who develop malignant melanoma following organ transplantation.

Since melanoma is considered a strongly immune-responsive tumor, one hypothesis proposes that immunosuppressive therapy in the post-transplant period may be the primary risk factor for melanoma [12]. Further, there is a belief that immunosuppressive therapy may also increase the aggressiveness of post-transplant melanoma, should it develop [13]. Postulated mechanisms suggest that T-cell depletion underlies the propensity for melanocyte atypia and dissemination [14]. Specifically, atypical melanocytes express a broad range of foreign antigens that serve as immune targets for T-cell-mediated destruction; however, such an immune response becomes significantly dampened in those receiving immunosuppressive therapy, which places patients at increased risk for melanoma with early and aggressive metastasis [14]. According to several studies, all immunosuppressant agents significantly increase the risk for melanoma, but the risk seems to be greatest in those who receive azathioprine, cyclosporine, or sirolimus [7,15]. One theory proposes the possibility that undiagnosed melanoma before transplant can easily go undetected, which then disseminates after immunosuppressive therapy is added in the post-transplant period [6]. Less likely is the possibility of melanoma transmission from organ donor to recipient, which has been reported but is considered exceedingly rare [16,17]. 

In the case presented herein, the patient was diagnosed with melanoma within three years of liver transplantation. This is fairly consistent with cases that report a markedly increased risk of melanoma within four years following transplant [18]. Despite surgical removal and appropriate follow-up based on screening guidelines, the patient still developed recurrent melanoma that was aggressive and diffusely metastasized at the time of recognition. Unfortunately, despite corticosteroids, radiation therapy, and modification of the immunotherapy regimen, the patient's condition worsened rapidly, and mortality occurred shortly after metastasis was detected. 

There are several interesting points that warrant consideration regarding this presentation. Firstly, it remains unknown whether this case resulted from a complication of immunosuppressive therapy. This specific patient was switched from tacrolimus to cyclosporine shortly after transplantation, and it is unclear whether this increased his risk of primary and recurrent melanoma. With that said, further research in the post-transplant population seems warranted to directly compare the relative risk of various T-cell immunomodulating agents in how they influence the development of melanoma. Additionally, the possibility of undiagnosed pre-transplant melanoma cannot be excluded, which raises the point of whether stringent pre-transplant melanoma skin mapping should be employed prior to organ transplantation. Furthering this point, current guidelines recommend yearly skin examination for patients at high risk for melanoma who meet any of the following criteria: (1) total nevus count above 50 and/or presence of large nevi; (2) personal history of skin cancer; (3) immunosuppression; and (4) very sun-sensitive individuals and those with "red hair phenotype" [19]. However, since the risk of mortality correlates with the stage of disease at initial presentation, guidelines may need to be modified to account for patients who have more than one of the aforementioned criteria. The patient discussed in this case developed skin cancer while receiving immunosuppression therapy, both of which are considered high-risk factors for developing recurrent melanoma. Nonetheless, in line with current guidelines, this patient continued to only undergo yearly skin screening examinations. Therefore, it is possible that if more stringent screening guidelines were established for such very-high-risk patients, the melanoma may be detected at an earlier stage.

## Conclusions

Recognizing and treating post-transplant complications is important for providing potentially lifesaving patient care. One such complication is that of malignant melanoma, which is associated with high mortality in the post-transplant population. Though the high prevalence of post-transplant malignant melanoma is well-documented, the mechanisms driving its incidence remain poorly understood. Additionally, although there are standardized guidelines for melanoma screening in high-risk patients, these screening guidelines are not modified for very-high-risk patients with multiple risk factors for melanoma. In such patients, screening modifications may be needed to identify melanoma early in its development. Therefore, in addition to further research to help elucidate the risks and mechanisms underlying the development of post-transplant melanoma, it seems that screening intervals should be more frequent in very-high-risk patients. Given the high mortality associated with advanced melanoma in the post-transplantation period, the implementation of more stringent screening guidelines may improve survival by aiding in the early detection of melanoma.
